# Using supernetworks to distinguish hybridization from lineage-sorting

**DOI:** 10.1186/1471-2148-8-202

**Published:** 2008-07-14

**Authors:** Barbara R Holland, Steffi Benthin, Peter J Lockhart, Vincent Moulton, Katharina T Huber

**Affiliations:** 1 Allan Wilson Centre, Institute of Fundamental Sciences, Massey University, Palmerston North, New Zealand; 2 Allan Wilson Centre, Institute of Molecular BioSciences, Massey University, Palmerston North, New Zealand; 3 School of Computing Sciences, University of East Anglia, Norwich, UK

## Abstract

**Background:**

A simple and widely used approach for detecting hybridization in phylogenies is to reconstruct gene trees from independent gene loci, and to look for gene tree incongruence. However, this approach may be confounded by factors such as poor taxon-sampling and/or incomplete lineage-sorting.

**Results:**

Using coalescent simulations, we investigated the potential of supernetwork methods to differentiate between gene tree incongruence arising from taxon sampling and incomplete lineage-sorting as opposed to hybridization. For few hybridization events, a large number of independent loci, and well-sampled taxa across these loci, we found that it was possible to distinguish incomplete lineage-sorting from hybridization using the filtered Z-closure and Q-imputation supernetwork methods. Moreover, we found that the choice of supernetwork method was less important than the choice of filtering, and that count-based filtering was the most effective filtering technique.

**Conclusion:**

Filtered supernetworks provide a tool for detecting and identifying hybridization events in phylogenies, a tool that should become increasingly useful in light of current genome sequencing initiatives and the ease with which large numbers of independent gene loci can be determined using new generation sequencing technologies.

## Background

In recent years there has been growing interest in the problem of building explicit models of reticulate evolution [1-12]. This work has to a large part been motivated by biological research highlighting the potential importance of hybridization in the origin of biotic diversity, biological invasion and rapid adaptation [13-27].

One simple and widely used approach for detecting hybridization has been to compare gene trees built from independent gene loci, and to consider gene tree incongruence as evidence for hybridization [1,28,29]. However, hybridization is not the only possible cause of gene tree incongruence. Other explanations include phylogenetic error [30], unrecognised gene duplication and loss [31], incomplete lineage-sorting [32] and lateral gene transfer [33].

In light of current genome sequencing efforts and the ease of sequencing large numbers of independent gene loci using new generation sequencing technologies, it is important to find ways to differentiate between various explanations of gene tree incongruence. Here we focus on distinguishing hybridization from incomplete lineage-sorting. In this regard, a helpful concept might be "principal trees", which are the trees displayed by a hybridization network (see the subsection *Simulations *for a more formal definition of principal trees). If a phylogeny contains no hybridization or lateral gene transfer, then the expectation is for one principal or "species" tree. However, if hybridization has occurred, then there will be multiple principal trees. In the absence of incomplete lineage-sorting, each principle tree will represent the evolutionary history for a large collection of loci, but where incomplete lineage-sorting occurs gene trees may differ from their underlying principal tree.

Here we investigate the potential of filtered Z-closure [34] and Q-imputation [35] supernetworks to distinguish phylogenetic signals arising from principal trees from phylogenetic signals caused by a combination of incomplete taxon-sampling and incomplete lineage-sorting in evolutionary histories involving hybridization. We consider these methods since they are not only designed to cope with conflicting phylogenetic signals, but also with data that current genome sequencing efforts can fail to gather (for example, for multi-gene data sets, such as those generated from expressed sequence tag (EST) databases, gene sequences are often missing for species of interest). In particular, using gene trees generated under a coalescent process, we test whether these methods can be used to filter out phylogenetic signals that do not correspond to edges in principal trees, signals that have the potential of confounding efforts to reconstruct complex phylogenies.

## Methods

### Overview

Analogous to the supertree framework [36] our input is a set of trees on overlapping but not necessarily identical taxa. We refer to the *complete taxa set *as the union of the taxa sets of the input trees; *complete splits *are bipartitions of the complete taxa set and *trivial splits *are splits where one part consists of precisely one element. Furthermore *partial trees *and *partial splits *are trees and splits on a subset of the complete taxa set. We denote a split of the taxa set X as A|B where A and B are both subsets of X, A ∪ B = X, and A ∩ B is the empty set.

Our overall approach is to first generate a collection of partial trees along a hybridization network in the presence of incomplete lineage-sorting (modelled by the coalescent process), to then apply a supernetwork method to this collection of partial trees to obtain a collection of complete splits, and to then apply a filter to reduce the complexity of this collection. The reduced collection of complete splits is then compared to the splits associated with the hybridization network to determine if they have been accurately recovered. We use this approach to study two supernetwork methods – Z-closure [34] and Q-imputation [35], and two types of filter – one counting-based and one homoplasy-based [37].

### Supernetwork methods

**Figure 1:**
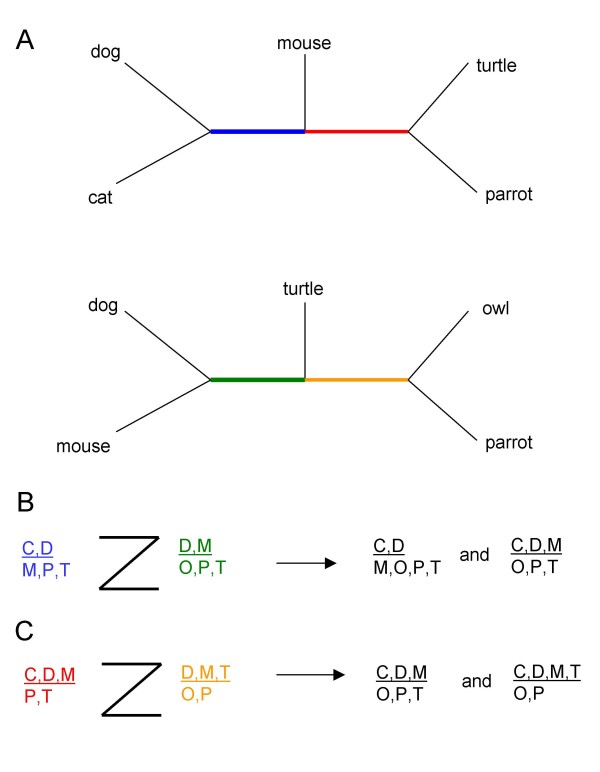
An example of two applications of the Z-rule, which underpins the Z-closure supernetwork method, where two partial splits displayed in the input trees (A) are extended to complete splits as shown in (B) and (C). The bold lines that form the 'Z' shape indicate that the intersection of the taxon sets is non-empty, eg in (B) {C,D}∩ {D,M} = {D}, {D,M}∩ {M,P,T} = {M}, {M,P,T}∩ {O,P,T} = {P,T}, but {C,D}∩ {O,P,T} = ∅ so the Z-rule can be applied.

We begin with a brief description of the two supernetwork methods that we considered. Supernetwork methods take as input a set of partial trees and produce a set of complete splits. Unlike the supertree framework, these splits need not be compatible, allowing possible conflict within the set of input trees to be represented. The first supernetwork method we consider, Z-closure, is underpinned by the Z-closure rule and is introduced in [34] and implemented in SplitsTree4 [38]. The method begins with a collection of partial trees from which a list of partial splits is obtained – each partial split coming from an edge in some partial tree in the list (see e.g. [8,34,38]). The Z-closure rule takes a pair of partial splits A|B and C|D, and if A ∩ C, B ∩ C and B ∩ D are all non-empty and A ∩ D is empty, then it replaces the partial splits A|B and C|D with the extended splits A| B ∪ D and A ∪ C | D (c.f. Figure 1). To ensure that as many partial splits in the list as possible are extended to complete splits, the rule is iteratively applied to the partial splits in the list by taking pairs of partial splits and either overwriting them with two splits on a more inclusive taxa set, or, if the rule does not apply, returning the same two partial splits. When the Z-closure rule can no longer be applied the method returns the list of complete splits that have been generated.

Note that the output of Z-closure is dependent on the order of elements in the list of partial splits, and so we repeat the procedure for 10 random orderings keeping a cumulative count of how many times each complete split appears. (Simulations indicate that this order dependence is not strong [5], so there would be little benefit in performing a larger number of random orderings.) Also note that the Z-closure implementation used for this paper differs slightly from that in SplitsTree4 in that it keeps track of multiple copies of partial splits and complete splits, as this information is required by the counting filter that we apply later.

The other supernetwork method we consider, Q-imputation [35], also uses partial trees as input but uses an alternative approach to generating complete splits that is based on the four-taxon subtrees (quartets) of the partial trees. For each partial tree with missing taxa – that is, taxa that are in the complete taxa set but not in the taxa set for that tree – the missing taxa are inserted in the tree. This is done by processing the missing taxa in a fixed order and placing each taxon within the partial tree to maximise the number of quartets that contain the missing taxon and are also quartets of the other partial input trees. Once all the trees have been completed the list of complete splits displayed by the completed trees is returned. (In the special case where all the trees are on identical taxa sets, the Q-imputation method reduces to the consensus network method [39]).

### Filtering methods

We apply two different kinds of filter to the lists of complete splits obtained from the two supernetwork methods, a homoplasy-based filter and a counting-based filter. The homoplasy filter [37] requires two user-defined parameters *x *and *y*. The level of homoplasy for each complete split and partial tree is determined by recoding the split as a binary character, reducing it to the same taxa set as the partial tree, and evaluating the number of character state changes required to explain the character on the partial tree (i.e. the parsimony score). Splits that have a parsimony score greater than a given number *x *in more than a given number *y *of the partial trees are filtered out. The counting filter has one user-defined parameter *n *and keeps the *n *splits that appear most frequently in the list of complete splits (ties are broken arbitrarily). Note that for Q-imputation this is equivalent to the filter described in [35] for some choice of threshold.

### Simulations

**Figure 2:**
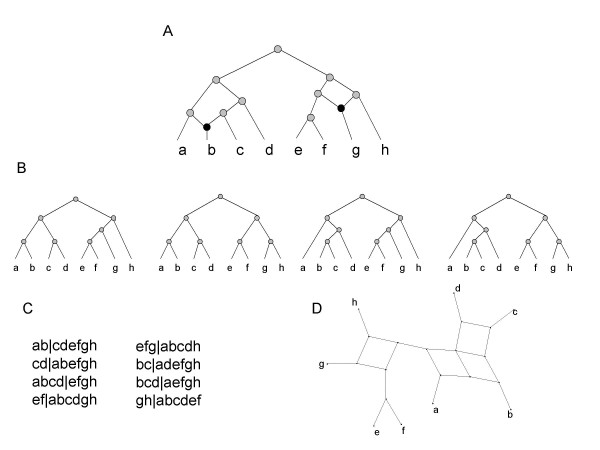
(A) A hybridization network (number 7 from Table 1) with two hybridization nodes. (B) The principal trees of the hybridization network – these are found by choosing a single parent at each hybridization node and then suppressing the resulting internal nodes of degree 2. (C) The splits associated with the hybridization network are those displayed by the principal trees in (B). (D) A split network displaying the splits in (C).

The starting point for each simulation is a hybridization network such as the one shown in Figure 2a. Formally such networks are rooted, leaf-labelled, directed-acyclic-graphs in which the nodes are of one of four types: nodes with in-degree 2 and out-degree 1 correspond to hybridizations; nodes with in-degree 1 and out-degree 2 correspond to speciation events; nodes with in-degree 1 and out-degree 0 correspond to the extant species; and one special node of in-degree 0 and out-degree 2 is the root. Such a network can be thought of as a collection of rooted principal trees: These trees are obtained by starting from the tips of the hybridization network (these are the nodes with in-degree 1 and out-degree 0) and choosing one of the two possible paths at each hybridization node that is encountered on the way towards the root. The set of principal trees consists of the trees possible to obtain in this manner (Figure 2b). This leads to a natural definition of the collection of splits associated with a hybridization network as being the union of the splits associated with each of the principal trees of the network (Figure 2c and 2d). We will refer to such splits as the true splits of the hybridization network. The purpose of the simulations is to assess if filtered supernetworks can identify the splits present in the principal trees of the hybridization network. To be successful these splits need to be distinguishable from those arising from incomplete lineage-sorting under the coalescent process.

**Figure 3:**
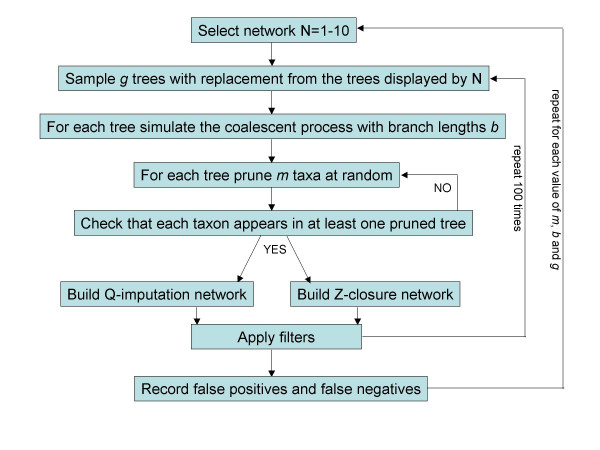
Flowchart indicating the steps used in the simulation study.

The main flow of our simulation is shown in Figure 3. Given a hybridization network, a collection of trees was created by sampling with replacement from the collection of principal trees (the same tree may appear multiple times). We used the software package COAL [32] to simulate trees according to the coalescent process given a principal tree with branch lengths specified in coalescent units (the number of generations divided by population size). We employed two different branch length settings. In each of the principal trees all branch lengths were either assigned coalescent units of 1 or all branch lengths were assigned coalescent units of 0.5. These choices for the branch length settings were also used in [32], a more sophisticated approach might be to assign branch lengths to the hybridization network itself and then have the principal trees inherit these branch lengths. We also simulated a situation where there were no lineage-sorting effects. In this case the only random aspect to the data generation is the choice of the principal trees. Each tree was then pruned of *m *taxa at random with the restriction that each taxon in the network must appear in at least one partial tree. These collections of partial trees were then used as input to each of the supernetwork methods. Note that, although COAL produces rooted trees, neither of the 2 supernetwork methods considered use any information about the root, so in effect they consider only the corresponding unrooted trees.

Accuracy of the supernetwork methods was determined by counting the number of false positive and false negative splits. A *false positive *is a split that is displayed by the supernetwork, but is not a true split of the hybridization network; a *false negative *is a split that is displayed by at least one of the principal trees of the hybridization network, but not displayed by the supernetwork. Note that these definitions differ from those used in [34] in that they measure accuracy with respect to an underlying hybridization network that has been used to generate the data.

We based our simulations on ten different hybridization networks each labelled by 8 taxa, and representing 0 to 3 hybridization events (Table 1). For each network the parameters in the simulation that varied were the number of trees (*g *= 5, 10, 15 or 20), the branch lengths used by COAL [32] (*b *= 8, 1, 0.5; where 8 represents the case where COAL was not used, i.e. there are no incomplete lineage-sorting effects) and the number of taxa missing from each tree (*m *= 0, 1, 2, or 3). For each parameter setting we made 100 replicate data sets, giving 6400 sets of partial trees for each of the ten hybridization networks.

**Table 1:** Hybridization networks used in simulations.

ID	H	S	Principle trees (given in Newick format)
1	0	5	(((a,b),(c,d)),((e,f),(g,h)));
2	1	8	(((a,b),(c,d)),((e,f),(g,h))); (((a,b),c),(((e,d),f),(g,h)));
3	1	7	(((a,b),(c,d)),((e,f),(g,h))); (((a,(b,d)),c),((e,f),(g,h)));
4	0	5	(((((((a,b),c),d),e),f),g),h);
5	1	7	(((((((a,b),c),d),e),f),g),h); ((((((a,c),(b,d)),e),f),g),h);
6	1	10	(((((((a,b),c),d),e),f),g),h); ((((((a,c),d),e),f),(g,b)),h);
7	2	8	(((a,b),(c,d)),((e,f),(g,h))); (((a,b),(c,d)),(((e,f),g),h));
			((a,((b,c),d)),((e,f),(g,h))); ((a,((b,c),d)),(((e,f),g),h));
8	2	9	(((a,b),(c,d)),((e,f),(g,h))); (((a,b),c),(((d,e),f),(g,h)));
			((((a,b),(g,h)),(c,d)),(e,f)); ((((a,b),(g,h)),c),((d,e),f));
9	3	9	((((a,b),(c,d)),(e,f)),(g,h)); ((((a,b),(c,d)),e),(f,(g,h)));
			(((a,b),(c,d)),((e,f),(g,h))); (((a,b),(c,d)),(e,(f,(g,h))));
			((((a,b),c),(d,(e,f))),(g,h)); ((((a,b),c),(d,e)),(f,(g,h)));
			(((a,b),c),((d,(e,f)),(g,h))); (((a,b),c),((d,e),(f,(g,h))));
10	3	24	((((b,e),(a,c)),((d,f),g)),h); (((b,(a,c)),((e,g),(d,f))),h);
			(((a,(b,e)),(((c,d),f),g)),h); (((a,b),(((c,d),f),(e,g))),h);
			(((a,c),((((b,e),d),f),g)),h); (((a,c),(((b,d),f),(e,g))),h);
			((a,((((b,e),(c,d)),f),g)),h); ((a,(((b,(c,d)),f),(e,g))),h);

The column H gives the number of hybridization events, and the column S gives the number of unique non-trivial splits contained in the principal trees.

We applied 4 different homoplasy filters to the lists of complete splits returned by Z-closure and Q-imputation:

• (HF1) keep only splits with no homoplasy (i.e. a parsimony score of 1) on all partial trees,

• (HF2) keep only splits with no homoplasy on 75% or more of the partial trees,

• (HF3) keep only splits with no homoplasy on 50% or more of the partial trees,

• (HF4) keep splits with a parsimony score of 2 or less on all partial trees.

We also applied the counting filter (CF) to both Z-closure and Q-imputation. For each hybridization network we selected *n *splits, where *n *was fixed to be the number of unique non-trivial splits associated with the principal trees of the network (Table 1).

## Results and Discussion

**Figure 4:**
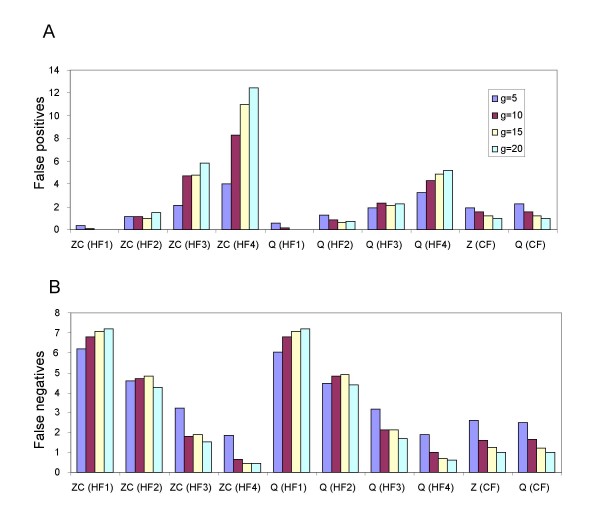
False positives (A) and false negatives (B) with increasing numbers of input trees for Z-closure (ZC) and Q-imputation (Q) keeping splits with no homoplasy on any tree (HF1), keeping splits with no homoplasy on 75% or more of the trees (HF2), keeping splits with no homoplasy on 50% or more of the trees (HF3), keeping splits with a homoplasy score of 1 or less on all of the trees (HF4), or keeping the 8 highest weight splits (CF) for hybridization network 7. Values are averages over the 12 combinations of coalescent branch length *b *and number of missing taxa *m*. The maximum possible number of false negatives for this hybridization network is 8.

**Figure 5:**
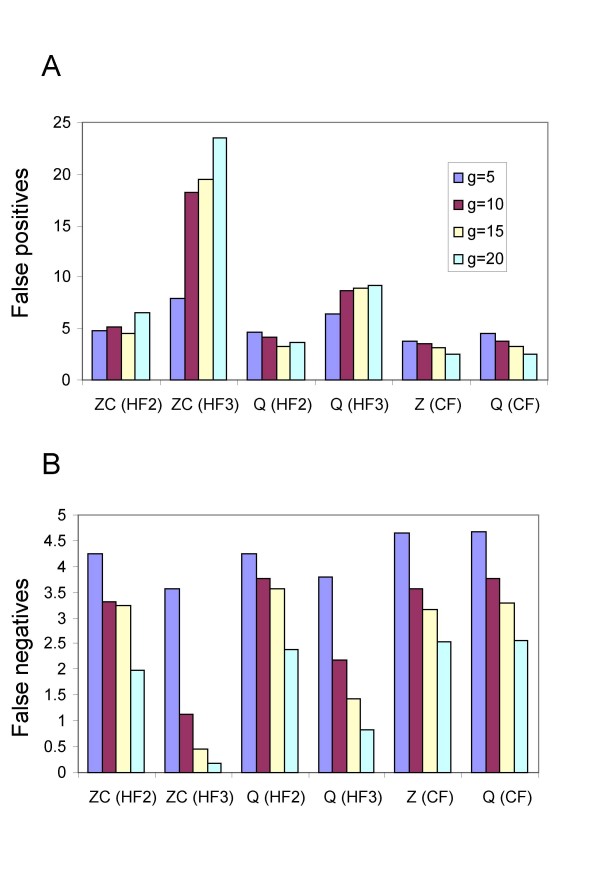
False positives (A) and false negatives (B) with increasing number of input trees for the highest setting of missing taxa (*m *= 3) and the smallest setting for coalescent branch lengths (*b *= 0.5) for hybridization network 7. Abbreviations are as descibed in Figure 4. The maximum possible number of false negatives for this hybridization network is 8.

**Figure 6:**
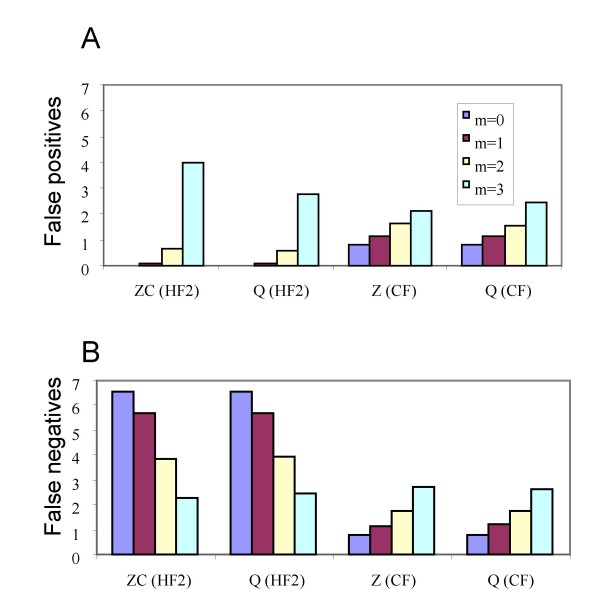
False positives (A) and false negatives (B) as the number of missing taxa *m *increases from 0 to 3 for hybridization network 7. Results are averaged over the 12 possible settings for number of gene trees *g *and coalescent branch lengths *b*. Abbreviations are as descibed in Figure 4. The maximum possible number of false negatives for this hybridization network is 8.

**Figure 7:**
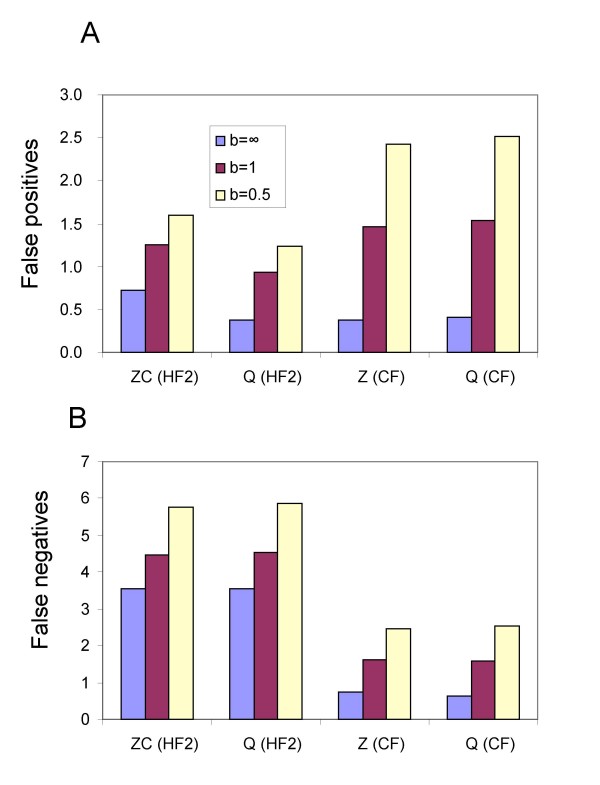
False positives (A) and false negatives (B) for the two different branch length settings using in the coalescent simulation (*b *= 0.5 and *b *= 1), and for the control without incomplete lineage-sorting (*b *= 8) for hybridization network 7. Results are averaged over the 16 possible settings for number of gene trees *g *and number of missing taxa *m*. Abbreviations are as descibed in Figure 4. The maximum possible number of false negatives for this hybridization network is 8.

Results were generated for each of the hybridization networks given in Table 1, but for brevity, in Figures 4, 5, 6, 7 and Table 2 we only show results for hybridization network 7 (the network shown in Figure 2a). Results for the other hybridization networks follow the same general trends [see Additional file 1].

**Table 2:** False positives for hybridization network 7 using the counting filter to select the 8 highest weight splits.

		Q-imputation					Z-closure			
		**m = 0**	**m = 1**	**m = 2**	**m = 3**		**m = 0**	**m = 1**	**m = 2**	**m = 3**
**b = ∞**	**g = 5**	0	0.2	0.64	2.18	**g = 5**	0	0.23	0.56	0.66
	**g = 10**	0	0.07	0.33	1.25	**g = 10**	0	0.17	0.56	1.26
	**g = 15**	0	0.08	0.25	0.86	**g = 15**	0	0.12	0.49	0.98
	**g = 20**	0	0.01	0.18	0.47	**g = 20**	0	0.04	0.29	0.76
										
		**m = 0**	**m = 1**	**m = 2**	**m = 3**		**m = 0**	**m = 1**	**m = 2**	**m = 3**
**b = 1**	**g = 5**	1.62	2.05	2.29	3.38	**g = 5**	1.61	1.97	2.25	2.25
	**g = 10**	0.68	1.44	1.98	2.69	**g = 10**	0.67	1.37	1.96	2.6
	**g = 15**	0.34	0.97	1.37	2	**g = 15**	0.33	0.84	1.43	2.1
	**g = 20**	0.18	0.74	1.05	1.93	**g = 20**	0.18	0.68	1.12	2.01
										
		**m = 0**	**m = 1**	**m = 2**	**m = 3**		**m = 0**	**m = 1**	**m = 2**	**m = 3**
**b = 0.5**	**g = 5**	2.85	3.24	3.74	4.56	**g = 5**	2.79	3.04	3.71	3.78
	**g = 10**	1.73	2.25	2.68	3.76	**g = 10**	1.71	2.18	2.64	3.56
	**g = 15**	1.3	1.67	2.24	3.28	**g = 15**	1.31	1.65	2.35	3.16
	**g = 20**	0.94	1.43	2.12	2.57	**g = 20**	0.95	1.33	2.05	2.54

### Filtering

Figure 4 shows the change in the average number of split false positives and false negatives with respect to the number of gene trees. The results are averaged over 100 repetitions and the 12 combinations of number of missing taxa *m *and coalescent branch lengths *b*.

As can be seen in Figure 4, the (HF1) filter is far too stringent in combination with either Z-closure or Q-imputation; it gives almost no false positives but false negatives increase with increasing *g *towards the maximum value of 8. The (HF4) filter is not stringent enough in combination with either Z-closure or Q-imputation; it gives almost no false negatives but false positives increase with increasing *g*. Moreover, (HF3) is ineffective in combination with Z-closure as the number of false positives increases with increasing number of partial trees, in combination with Q-imputation the average number of false positives stays close to 2 for all values of *g*. (HF2) is the most effective of the homoplasy filters, as for both Z-closure and Q-imputation both types of errors either decrease or stay reasonably constant with increasing *g*, a property that we would expect any filtered supernetwork method to satisfy. The counting filter also displays this property for both Z-closure and Q-imputation; both false positives and false negatives decrease with increasing number of input trees.

Figure 5 is similar to Figure 4 except that rather than averaging over all values of *b *and *m *we focus on the difficult case with the highest number of missing taxa and the most incongruence generated by incomplete lineage-sorting (*m *= 3, *b *= 0.5). While all the filtered supernetwork methods shown in Figure 5 control false negatives, false positives increase with increasing *g *for both Z-closure and Q-imputation using (HF3).

For the rest of this section, we restrict our attention to the best homoplasy filter (HF2) and the counting filter (CF).

### Missing taxa

Figure 6 shows the trends in the number of false positives and false negatives as the number of missing taxa per tree, *m*, increases from 0 to 3. Results are averaged over the 12 possible settings for number of partial trees *g *and coalescent branch lengths *b*. The (HF2) and (CF) filters exhibit very different behaviour. For (HF2) as *m *increases the number of false positives increases, in particular going from 2 to 3 missing taxa produces a dramatic increase. Conversely the number of false negatives decreases, presumably due to the fact that as the number of missing taxa gets large more splits meet the requirement of the filter. This effect was not observed for (CF) where the total number of splits is capped; here both false positives and negatives increase with growing *m*.

### Incomplete lineage-sorting

Recall that the parameter *b *affects the probability that the trees generated by COAL [32] will match the principal tree sampled from the hybridization network, *b *= 8 corresponds to trees that match exactly. Figure 7 shows the trends in the number of false positives and false negatives for different values of *b*. Results are averaged over the 16 possible settings for the number of partial trees *g *and the number of missing taxa *m*. As expected, both methods and filters perform better when *b *is large.

### Overall performance

Table 2 shows the number of false positives for hybridization network 7 (Figure 2a), which has 2 hybridization events and 8 true splits, for *m *= 0, 1, 2 or 3, *b *= 0.5, 1 or 8, and *g *= 5, 10, 15 or 20 for both Z-closure and Q-imputation with (CF). If two out of three of the conditions (*g*, *m*, or *b*) are favourable (i.e. many input trees, few missing taxa, and the probability that the input trees are congruent with the principal trees is high) then both methods work well. However if two or three of the conditions are unfavourable then both methods start to break down.

**Figure 8:**
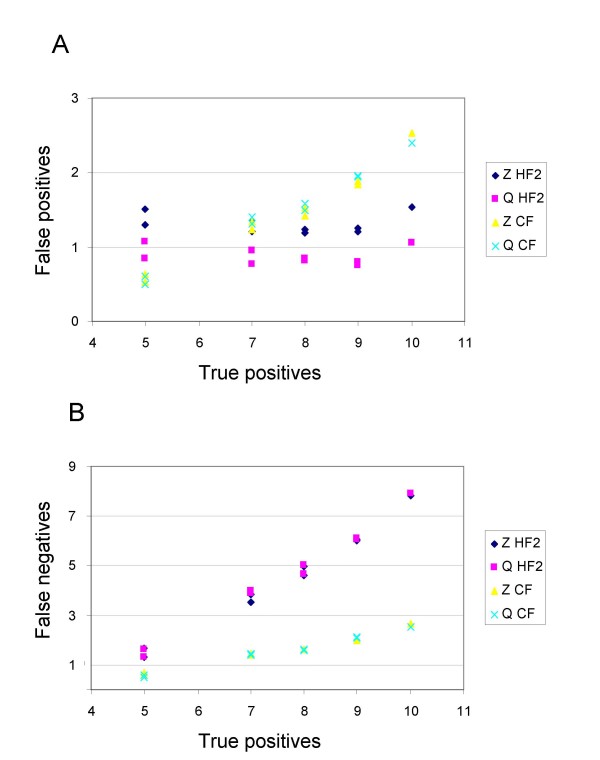
False positives (A) and false negatives (B) averaged over 48 combinations of number of missing taxa *m*, number of gene trees *g*, and coalescent branch lengths *b *versus the number of true splits for hybridization networks 1–9. Note that the number of true splits is the maximum possible number of false negatives.

Figures 8a and 8b show the average number of false positives and false negatives respectively (averaged over *m*, *g*, and *b*) versus the number of true splits for hybridization networks 1–9. Hybridization network 10 is not shown in the figures, as it is an outlier with 24 true splits, but results for this network follow the same trends as the other hybridization networks. For (CF) both types of errors increase slowly with increasing number of true splits. For (HF2) false positives appear fairly constant, but false negatives increase linearly with a slope close to one with increasing number of true splits.

## Conclusion

We have evaluated the potential of Z-closure and Q-imputation filtered supernetworks to identify splits belonging to the sets of principal trees associated with hybridization networks. We have found that this approach can recover these splits when there are few hybridization events. However, our results imply that (1) if gene trees have many missing taxa then many gene trees are required; (2) if the gene trees are frequently incongruent with the principal trees of the hybridization network due to incomplete lineage-sorting then a large number of near complete gene trees is required; (3) and if there are few gene trees available they need to be both near complete and highly congruent with the principal trees.

In our simulations the counting filter picked the n best-supported splits, where n was chosen to be the known number of true non-trivial splits. Of course with real data n will not be known, although in practice n could be chosen by, for example, greedily introducing splits with highest support as long as the corresponding network does not become too complex to easily interpret. Approaches to do this are described in [40] for consensus networks. Note that by increasing n the risk of introducing false positive splits is increased, although the risk of failing to identify true positive splits is reduced.

Despite these limitations, with the potential now of obtaining large numbers of splits from independent gene loci using new generation sequencing technologies, our findings may nevertheless be applicable for tree-like phylogenies where some degree of hybridization is inferred [41]. In such cases, filtered supernetworks can be used to identify the true splits of the underlying hybridization network. Once these are obtained, the method of [5] can be used to convert the split system into a hybridization scenario.

One of our most interesting findings is that the choice of whether to use Z-closure or Q-imputation seems to have much less impact on accuracy with regards to recovering the splits in the underlying hybridization network than the choice of filter. For both Q-imputation and Z-closure the counting filter (CF) has the desirable property that as the amount of data increases (more genes or more complete gene trees) the rate of both false positives and false negatives goes down. Several settings were tried for the homoplasy-based filter (HF1 – HF4). HF1 was too stringent, and HF3/HF4 tended to either suffer from increasing false positives or increasing false negatives as the number of gene trees increased. HF2 gave the best compromise between these extremes.

Using the HF2 filter, we found that Z-closure had a higher false positive rate than Q-imputation over a range of parameter combinations (Figure 8A). One explanation might be that Z-closure can potentially generate more splits than Q-imputation. For example, given *g *fully resolved gene trees on 8-*m *taxa (*m *= 1, 2, 3), Q-imputation can generate at most 5*g non-trivial complete splits, whereas Z-closure can produce at most 10*(5-*m*)*g non-trivial complete splits (where 10 is the number of random orderings). Hence the maximum number of splits that Z-closure could generate decreases as *m *grows, whereas the number of splits that Q-imputation could generate stays constant. What we observe for both methods and filters is that false positives increase with increasing *m *(Figure 6). Therefore, the maximum number of splits that Z-closure and Q-imputation could generate does not appear to explain the difference in false positive rates. We think a more likely explanation is that Q-imputation places missing taxa in such a way as to maximize agreement with the input trees, hence tending to produce multiple copies of the same splits. Conversely, Z-closure aims to find all possible complete splits that can be derived by extending partial splits using the Z-closure rule, a process that can yield many different splits. Hence we expect that Q-imputation would be likely to generate fewer false positives than Z-closure in general. This difference is not greatly reduced by HF2 as, in contrast to CF, it does not place a cap on the total number of splits.

We found that HF2 resulted in more false negatives than CF (Figure 8B). This may be due to the fact that this filter only selects splits that have no homoplasy when restricted to 75% of the input trees. Since the principal trees are obtained from a network, they can be different and in some cases may only agree on a small number of edges. Even a true split may have a high homoplasy score when restricted to a particular principal tree. In contrast, CF only selects splits that occur with high frequency, irrespective of whether they are in agreement with any of the input trees.

Although all the trees used in our simulations were fully resolved, both supernetwork methods considered here can be applied to partially resolved trees. Thus, when inferring gene trees to be used as input to a supernetwork method, it would probably be a reasonable approach to only retain those edges in the estimated gene trees that have high support (e.g. bootstrap support or posterior probability higher than some cut-off value).

In cases where there are many hybridization events, especially between individuals that are not closely related, there will be many principal trees and corresponding splits (as in hybridization network 10). Many of these splits will occur at low frequencies making them hard to distinguish from phylogenetic error. This means that phylogenetic inference will be limited, as gene-tree incongruence will be extensive. In such cases, rather than attempt to reconstruct a hybridization network, it may be more appropriate to formulate objective tests to better understand the complexity of the data and the extent to which hybridization contributes to this complexity. Joly, McLenachan and Lockhart (submitted manuscript) have recently proposed such a test.

An unexplored idea worthy of study is the investigation of model-based, rather than combinatorial, methods of filtering. One approach might be to consider posterior probability distributions on species trees [42]. It will be interesting to investigate whether such posterior distributions can also be analysed for evidence of distinct principal trees in cases where evolutionary relationships are complex.

## Authors' contributions

BRH developed and applied the simulation scheme, implemented the modified Z-closure method, homoplasy filter and counting filter, and contributed to writing the ms, especially the methods and results section. SB conducted initial simulations with Z-closure. PL ensured biological relevance, and contributed to writing the ms, especially the introduction and conclusions. VM ensured mathematical correctness and developed the overall concept. KH ensured mathematical correctness and developed the overall concept, contributed to writing the ms, especially the methods and results section.

## References

[B1] Linder CR Rieseberg L Reconstructing patterns of reticulate evolution in plants Amer J Botany 2004 91 1700 1708 18677414 PMC2493047

[B2] Gusfield D Eddhu S Langley CH Optimal, efficient reconstruction of phylogenetic networks with constrained recombination J Bioinform Comput Biol 2004 2 1 173 213 15272438 10.1142/s0219720004000521

[B3] Nakhleh L Warnow T Linder CR St. John K Reconstructing reticulate evolution in species–theory and practice J Comput Biol 2005 12 6 796 811 16108717 10.1089/cmb.2005.12.796

[B4] Jansson J Sung W Inferring a level-1 phylogenetic network from a dense set of rooted triplets Springer Lecture Notes in Computer Science 2004 3106 462 471

[B5] Huson DH Kloepper T Lockhart PJ Steel M Miyano S Reconstruction of reticulate networks from gene trees RECOMB 2005 LNBI 3500 2005 Springer-Verlag Heidelberg 233 249

[B6] Song YS Hein J Constructing minimal ancestral recombination graphs J Comput Biol 2005 12 2 147 169 15767774 10.1089/cmb.2005.12.147

[B7] Baroni M Semple C Steel M Hybrids in real time Syst Biol 2006 55 46 56 16507523 10.1080/10635150500431197

[B8] Semple C Steel M Phylogenetics 2003 Oxford: Oxford University Press

[B9] Huber KT Oxelman B Lott M Moulton V Reconstructing the evolutionary history of polyploids from multi-labelled trees Mol Biol Evo 2006 23 1784 1791 10.1093/molbev/msl045 16798795

[B10] Willson S Unique determination of some homoplasies at hybridization events Bull Math Biol 2007 69 1709 1725 17318673 10.1007/s11538-006-9187-4

[B11] Cardona G Rosselló F Valiente G Tripartitions do not always discriminate Phylogenetic Networks Math Biosci 211 356 370 18177903 10.1016/j.mbs.2007.11.003

[B12] van Iersel L Keijsper J Kelk S Stougie L Constructing level-2 phylogenetic networks from triplets 2007 arXiv:0707.2890v1 [q-bio.PE]

[B13] Linnaeus C Species plantarum, exhibentes plantas rite cognitas, ad genera relatas, cum differentiis specificis, nominibus trivialibus, synonymis selectis, locis natalibus, secundum systema sexuale digestas 1753 Holmiae: Impensis Laurentii Salvii [L. Salvius, Stockholm]

[B14] Anderson E Hybridization of the habitat Evolution 1948 2 1 9

[B15] Shaw KL Stebbins GL The role of hybridization in evolution Proc Am Philos Soc 1959 103 231 251

[B16] Ehrendorfer FR Differentiation-hybridization cycles and polyploidy Cold Spring Harb Symp Quant Biol 1959 24 141 152 13819584 10.1101/sqb.1959.024.01.014

[B17] Rattenbury JA Cyclic hybridization as a survival mechanism in the New Zealand forest flora Evolution 1962 16 348 363

[B18] Levin DA Hybridization and evolution – a discussion Taxon 1970 19 167 171

[B19] Grant V Plant speciation 1981 2 New York: Columbia University Press

[B20] Arnold ML Natural hybidization and evolution 1997 New York: Oxford University Press

[B21] Barrier M Baldwin BG Robichaux RH Purugganan MD Interspecific hybrid ancestry of a plant adaptive radiation: allopolyploidy of the Hawaiian Silversword alliance (Asteraceae) inferred from floral homeotic gene duplications Mol Biol Evol 1999 16 8 1105 1113 10474905 10.1093/oxfordjournals.molbev.a026200

[B22] Ellstrand NC Schierenbeck KA Hybridization as a stimulus for the evolution of invasivness in plants Proc Natl Acad Sci USA 2000 97 7043 7050 10860969 10.1073/pnas.97.13.7043 PMC34382

[B23] Barton NH The role of hybridization in evolution Molecular Ecology 2001 10 551 568 11298968 10.1046/j.1365-294x.2001.01216.x

[B24] Rieseberg LH Raymond O Rosenthal DM Lai Z Livingstone K Nakazato T Durphy JL Schwarzbach AE Donovan LA Lexer C Major ecological transitions in annual sunflowers facilitated by hybridization Science 2003 301 1211 1216 12907807 10.1126/science.1086949

[B25] Seehausen O Hybridization and adaptive radiation Trends Ecol Evol 2004 19 198 207 16701254 10.1016/j.tree.2004.01.003

[B26] Wissemann V Plant evolution by means of hybridization Systematics and Biodiversity 2007 5 243 253

[B27] Mallet J Hybrid speciation Nature 2007 446 279 283 17361174 10.1038/nature05706

[B28] Palmer JD Shields CR Cohen DB Orton TJ Chloroplast DNA evolution and the origin of amphidiploid Brassica species Theor Appl Genet 1983 65 181 189 10.1007/BF00308062 24263412

[B29] Sang T Zhong Y Testing hybridization hypotheses based on incongruent gene trees Syst Biol 2000 49 3 422 434 12116420 10.1080/10635159950127321

[B30] Cusimano N Zhang L-B Renner SS Revaluation of the cox1 group I intron in Araceae and Angiosperms suggests a history dominated by loss rather than horizontal transfer Mol Biol Evol in press 10.1093/molbev/msm241 18158323

[B31] Hallet M Lagergren J Tofigh A Simultaneous identification of duplications and lateral transfers Proceedings of the eighth annual international conference on Resaerch in computational molecular biology San Diego; ACM 2004 347 356

[B32] Degnan JH Salter LA Gene tree distributions under the coalescent process Evolution 2005 59 1 24 37 15792224

[B33] Doolittle WF Bapteste E Pattern pluralism and the Tree of Life hypothesis Proc Natl Acad Sci U S A 2007 104 7 2043 2049 17261804 10.1073/pnas.0610699104 PMC1892968

[B34] Huson DH Dezulian T Kloepper TK Steel MA Phylogenetic supernetworks from partial trees IEEE Trans Comput Biol Bioinform 2004 1 151 158 10.1109/TCBB.2004.44 17051697

[B35] Holland BR Conner G Huber K Moulton V Imputing supertrees and supernetworks from quartets Syst Biol 2007 56 1 57 67 17366137 10.1080/10635150601167013

[B36] Bininda-Emonds ORP The evolution of supertrees Trends Ecol Evol 2004 19 315 22 16701277 10.1016/j.tree.2004.03.015

[B37] Huson D Steel MA Whitfield J Buecher P, Moret BME Reducing distortion in phylogenetic networks Proceedings of WABI (Workshop on algorithms in bioinformatics) 2006 Springer-Verlage Berlin Heidelberg Lecture Notes in Bioinformatics 475:150–161

[B38] Huson DH Bryant D Application of phylogenetic networks in evolutionary studies Mol Biol Evol 2006 23 2 254 267 16221896 10.1093/molbev/msj030

[B39] Holland BR Huber KT Moulton V Lockhart PJ Using consensus networks to visualize contradictory evidence for species phylogeny Mol Biol Evol 2004 21 7 1459 1461 15084681 10.1093/molbev/msh145

[B40] Holland BR Delsuc F Moulton V Visualizing conflicting evolutionary hypotheses in large collections of trees using consensus networks Syst Biol 2005 54 1 66 76 15805011 10.1080/10635150590906055

[B41] Lockhart PJ McLenachan PA Havell D Glenny D Huson D Jensen U Phylogeny, dispersal and radiation of New Zealand alpine buttercups: molecular evidence under split decomposition Ann Missouri Bot Gard 2001 88 458 477

[B42] Edwards SV Liu L Pearl DK High-resolution species trees without concatenation Proc Natl Acad Sci U S A 2007 104 14 5936 5941 17392434 10.1073/pnas.0607004104 PMC1851595

